# Supplementary material for: SyNDI: synchronous network data integration framework
Source: BMC Bioinformatics. 2018 Nov 6;19:403. doi: 10.1186/s12859-018-2426-5 (PMC6219086; doi:10.1186/s12859-018-2426-5)
Supplement: Supplementary file 5 — Files of the example presented in Additional file 4. This additional file is a zip package that contains all files used in Additional file 4. Detailed descriptions of each of these files are presented in a README file included in this zip package. (GZ 30411 kb) [file 12859_2018_2426_MOESM5_ESM.gz › SyNDITools/Meme2Fimo/meme_4.9.0_4.tar.gz!/meme_4.9.0/website/html/mhmm-overview.in]

Meta-MEME v3.3


> ### Overview
>
> ---
>
> ### Introduction
>
> Meta-MEME is a software toolkit for building and using motif-based
> hidden Markov models of biological sequences. The input to
> Meta-MEME is a set of similar DNA or protein sequences, as well as
> a set of motif models discovered by MEME. Meta-MEME combines these models
> into a single, motif-based hidden Markov model and uses this model
>  to search a sequence database for homologs.
>
> ### Program inputs
>
> Meta-MEME takes as input two files:
>
> - The **sequence file** contains a set of similar DNA or
>   protein sequences that you are interested in modeling. Typically,
>   these will be sequences that are homologous to one another or that
>   share homologous domains. The sequences can be in various formats.
> - The **MEME motif models** are based upon the sequence file.
>   A MEME motif model is a position-specific scoring matrix; i.e., a
>   matrix in which position (i,j) contains the probability that
>   residue i will appear in position j in the motif. MEME motifs are
>   gapless, so the model contains no gap opening or extension
>   penalties. MEME motif models can be generated by the MEME web server
>   at the San Diego Supercomputer Center. Meta-MEME requires the MEME model
>   in HTML format.
>
> ### Step one: Building a model
>
> During the first, model-building step, Meta-MEME takes the
> single-motif models from the given MEME file and combines them into
> a motif-based hidden Markov model (HMM). An HMM is a generalization
> of a finite state machine in which each state in the model
> corresponds to a single residue. Probability distributions at each
> state represent the probabilities of point mutations; transition
> probabilities among states allow for insertions, deletions and, for
> some model topologies, repeated or shuffled domains.
>
> Meta-MEME's hidden Markov models differ from standard HMMs such
> as the ones produced by SAM and
> HMMER in that Meta-MEME's
> models are motif-based. Thus, the non-motif spacer regions are
> modelled imprecisely, thereby significantly reducing the number of
> parameters that need to be learned. This reduction in parameter
> space allows Meta-MEME models to be accurately trained from smaller
> sets of sequences.
>
> In constructing the HMM, Meta-MEME uses information about the
> order and spacing of motifs within the family.
> By default, Meta-MEME builds a model
> with a linear topology, in which the motifs are arranged like beads
> on a string.
> It is also possible to request that Meta-MEME build a model in
> which every motif is connected to every other motif. This
> completely connected topology allows for the accurate modeling of
> families containing repeated or shuffled elements.
>
> ### Step two: Homology detection
>
> A Meta-MEME
> model can be used to search a sequence database for homologs. The
> homology detection algorithm assigns to each sequence in the
> database a score that is proportional to the probability that the
> sequence was generated by the given model. Meta-MEME can compute
> two types of scores: the Viterbi score is the probability
> associated with the single path through the model that is most
> likely to have generated the given sequence; the total probability
> score is the sum of the probabilities of all possible paths through
> the model. By default, Meta-MEME computes the Viterbi score, since
> this score is less computationally expensive to compute. It is an
> open question whether Viterbi or total probability scoring produces
> better homology detection performance. You may request either or
> both types of scores.
>
> Both Viterbi and total probability scores are reported as
> log-odds scores in bits. An odds score is the ratio of the score of
> the sequence with respect to the foreground model versus the score
> of the sequence with respect to the background model. The log-odds
> score is the log (in base 2) of this ratio. In Meta-MEME, the
> foreground model is a motif-based HMM, and the background model is
> a simple linear HMM that roughly captures the features of a typical
> sequence. If the family in question is small relative to the size
> of the database being searched, then the odds score is
> approximately equivalent to the likelihood that the sequence
> belongs to the family in question divided by the likelihood that it
> does not. Hence, an odds score of 1 (or a log-odds score of 0)
> implies equal likelihood that the sequence is a family member or is
> not.
>
> The threshold for statistical significance of an odds score
> depends upon the expected number of family members in the database.
> A sequence can be safely deemed a family member if its odds score
> is greater than the ratio of the total number of sequences in the
> database over the expected number of family members. For example,
> if you are searching a database of 36000 sequences for a family
> containing approximately 100 sequences, then any sequence that
> receives an odds score of (36000 / 100) = 360 or higher likely
> belongs to the family. Since Meta-MEME reports log-odds scores,
> this threshold corresponds to log2(360) = 8.5.
>
> ---
>
> Return to the Meta-MEME home page.
>
> *Please send comments and questions to:
> @METAMEME\_CONTACT@.*
